# Overfishing and the Replacement of Demersal Finfish by Shellfish: An Example from the English Channel

**DOI:** 10.1371/journal.pone.0101506

**Published:** 2014-07-10

**Authors:** Carlotta Molfese, Doug Beare, Jason M. Hall-Spencer

**Affiliations:** 1 Marine Biology and Ecology Research Centre, Plymouth University, Plymouth, United Kingdom; 2 Worldfish, Batu Maung, Penang, Malaysia; University of Glasgow, United Kingdom

## Abstract

The worldwide depletion of major fish stocks through intensive industrial fishing is thought to have profoundly altered the trophic structure of marine ecosystems. Here we assess changes in the trophic structure of the English Channel marine ecosystem using a 90-year time-series (1920–2010) of commercial fishery landings. Our analysis was based on estimates of the mean trophic level (mTL) of annual landings and the Fishing-in-Balance index (FiB). Food webs of the Channel ecosystem have been altered, as shown by a significant decline in the mTL of fishery landings whilst increases in the FiB index suggest increased fishing effort and fishery expansion. Large, high trophic level species (e.g. spurdog, cod, ling) have been increasingly replaced by smaller, low trophic level fish (e.g. small spotted catsharks) and invertebrates (e.g. scallops, crabs and lobster). Declining trophic levels in fisheries catches have occurred worldwide, with fish catches progressively being replaced by invertebrates. We argue that a network of fisheries closures would help rebalance the trophic status of the Channel and allow regeneration of marine ecosystems.

## Introduction

### Effects of overfishing on marine trophic structure

The field of historical marine ecology has introduced a different perspective to our understanding of marine ecosystems; it has revealed that overfishing has had profound effects on coastal ecosystems worldwide for centuries [1], [2]. The historical response to overfishing is an increase in fishing effort, an expansion to new and deeper grounds and a shift to new target species [3]. In the last decade, fisheries have shifted towards smaller, lower-trophic level species as large predatory species with a higher economic value had been depleted [4]. This phenomenon, known as “fishing down marine food webs” was first described by [5] in 1998: they demonstrated a decline in the trophic level of global fisheries landings from 3.3 units in the early 1950s to 3.1 in 1994. Studies performed independently from commercial catch data on smaller, regional scales over the last decades have shown even more rapid declines in trophic level (Table 1).

**Table 1:** Instances of “Fishing Down Marine Food Web” across the globe, showing rates of decline in mean trophic level (mTL).

Country/Area	Period	mTL decline	Source
Cuba EEZ	1960–1995	0.10 decade^−1^	[14]
Canada (West and East coast)	1950–1997 and1873–1996	0.03–0.1 decade^−1^	[15]
Celtic Sea	1982–2000	0.04 year^−1^ (ICES catch data) and 0.03 year^−1^(scientific survey)	[16]
Thailand	1965–1997	0.05–0.09 decade^−1^	[17]
Iceland	1918–1999	0.06 decade^−1^	[18]
Chile	1979–1999	0.175 decade^−1^	[19]
Greece	1950–2001	0.02 decade^−1^	[20]
Indian States and Union Territories	1950–2000	0.058 decade^−1^	[21]
Argentinean-Uruguayan Common Fishing Zone(AUCFZ)	1989–2003	0.03 year^−1^	[22]
Portugal	1970–2006	0.005 year^−1^	[23]
Brazil	1978–2000	0.16 decade^−1^	[24]

Fisheries typically remove top predators first and as a result their direct competitors and prey are able to prosper, affecting the overall productivity and ecological stability of the ecosystem [1]. Severe declines in the populations of major predator species have now been reported around the world [6], [7]. Overexploitation of a species can have cascading effects and have the potential to trigger regime shifts altering the ecological function of marine systems [8], [9]. In many instances, the decline of finfish species has been followed by an increase in their invertebrate prey [10], [11] and although new and economically viable fisheries have developed for these new target species, concerns have been raised about their long-term sustainability as well as shifts towards homogenized, simplified ecosystems [12], [13].

**Figure 1:**
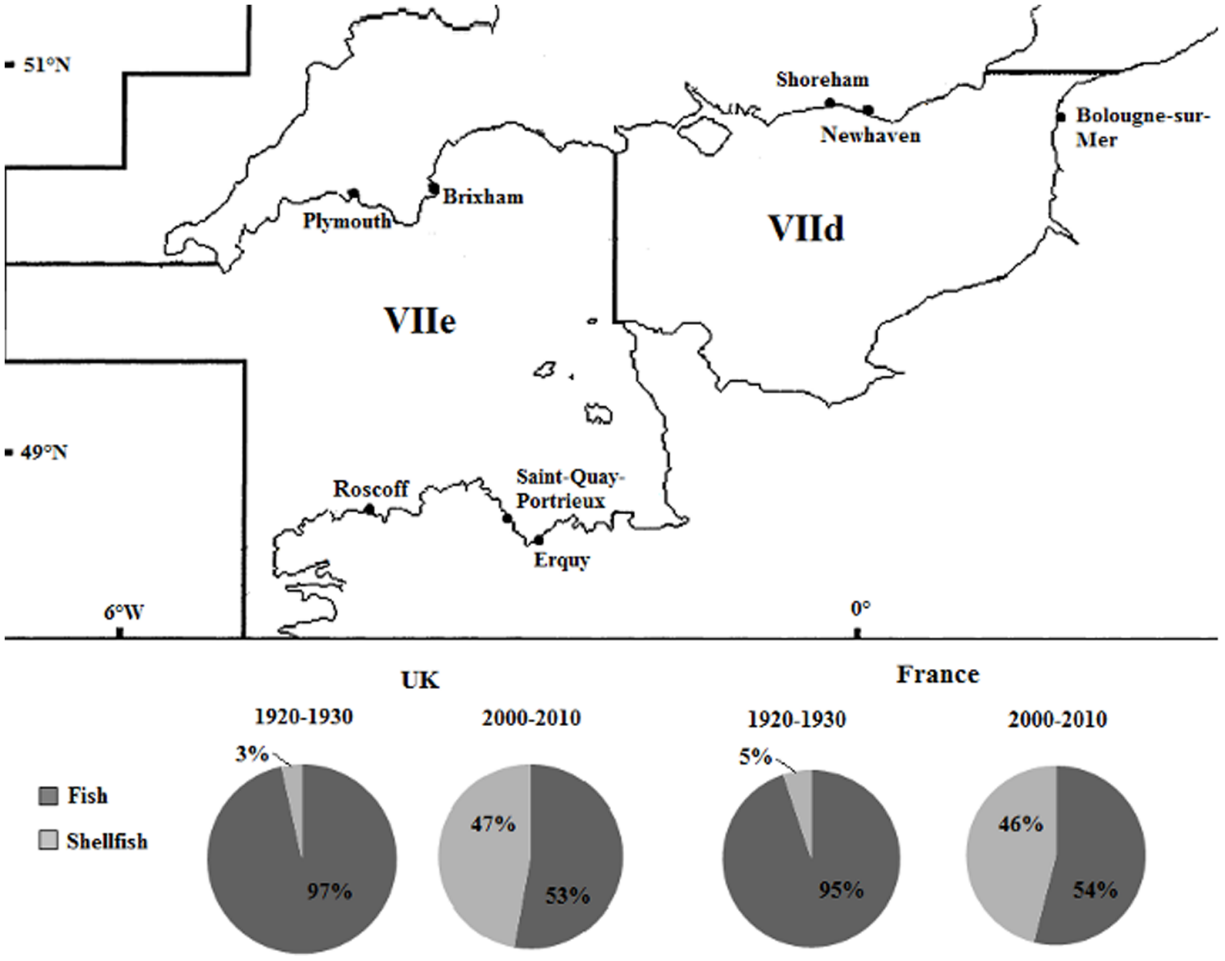
Major English Channel fishing ports by landings value in 2010, ICES areas VIIe and VIId. Data sourced from MMO and France AgriMer. Pie charts show the proportions of fish and shellfish landed by the UK and French fishing fleets for the period 1920–1930 and 2000–2010.

In the present study, we used a 90-year dataset of international catch statistics from the English Channel marine ecosystem, a region that has numerous important fishing ports and where finfish landings now make up a far smaller proportion of the catch than they did historically (Figure 1). This dataset spans a period of intensive fishing which we use to assess whether there has been a trend for ‘fishing down’ food webs in a region where it has not been reported before. Finally, we discuss the way forwards to improve fisheries sustainability using area closures to aid recovery of marine ecosystems.

### The English Channel

The English Channel (‘La Manche’ in French) is a narrow strait between England and France (Figure 1). It covers 75,000 km^2^ and narrows to *ca* 30 km wide at its easternmost point; the Channel is relatively shallow, with an average depth of around 100 m in the west gradually decreasing to 40 m depth in the east [25]. The western Channel is influenced greatly by Atlantic water while the eastern part receives large freshwater inputs from coastal rivers, this gradation from oceanic to neritic waters forms a biogeographical transition zone with a variety of both boreal/cold temperate and warm temperate organisms [26].

Today the UK and France account for 98% of the total landings from the Channel [27]: around 25,884 t and 68,732 t of finfish and 26,605 t and 48,871 t of invertebrate were landed in 2010 in the UK and France respectively. Fishing has exerted pressure in these waters since the Middle Ages [25], [26] but at the turn of the 20^th^ century fishing effort increased substantially due to the advent of motorised fishing vessels. Monitoring of fish populations has revealed reductions in mean length and length at maturity of demersal communities [28]. Moreover, large and slow-growing species have decreased dramatically in the region over the last century including angel shark (*Squatina squatina*, Squatinidae) and common skate (*Raja batis,* Rajidae) which is now commercially extinct in the Channel; whereas small, commercially undesirable species such as the small-spotted catshark (*Scyliorhinus canicula,* Scyliorhinidae) have increased in abundance [29], [30].

## Materials and Methods

### Fishing down marine food webs

The trophic level (TL) of an organism denotes its position within a foodweb and it can be estimated from diet observations, nitrogen isotope measurements or models of trophic fluxes from the equation:
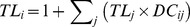
(1)Where *TL_j_* is the ‘non-integer’ trophic level of prey *j* and *DC_ij_* is the fraction of *j* in the diet of *i* [31]. The Marine Trophic Index (MTI), which corresponds to the mTL of fishery landings, was developed to describe the structure of an ecosystem resulting from a fishery-induced depletion of its components and can be computed for any year *y*:
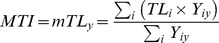
(2)Where *Y_iy_* is the catch of species *i* in year *y*, and *TL_i_* is defined as in Equation 1 [32].

Additionally, [31] developed an index to assess if changes in the mTL were compensated by changes in catches. This is because biological production is higher at lower TL, hence an inevitable consequence of ‘fishing down the food web’ will, ironically, be greater biological production potentially being available to the fisheries, and any decline in TL should be accompanied by an ‘ecologically appropriate’ increase in the overall biomass of catches. Hence, the Fishing-in-Balance (FiB) index:

  Will maintain a value of zero when a decrease or increase in TL is accompanied by an ecologically balanced increase or decrease in catches;
  Will increase (>0) if bottom-up effects have occurred or if the fishery has expanded beyond its traditional ground;
  Will decrease (<0) if the fishery has contracted geographically or if it has taken so much biological productivity from the ecosystem that has impaired its natural functioning.

It is defined for any year *y* by:
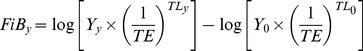
(3)Where *Y_y_* is the catch at year *y*; *TL_y_* is the trophic level of the catch in year *y*; *Y_0_* and *TL_0_* are the catch and trophic level of the catch at the beginning of the series analysed and TE is the energy transfer efficiency between TL estimated to be 0.1 ( = 10%) in several marine ecosystem studies [32].

### International fishery landings

Catch statistics for the English Channel were obtained from ICES (International Council for the Exploration of the Sea) [33] and comprised two datasets; Historical catch statistics (1903–1949) and Official catch statistics (1950–2010). The English Channel consists of West and East Channel areas defined as divisions VIIe and VIId respectively (Figure 1). The statistics represent the live weight equivalent of landings and so do not include discards. In our analyses we excluded all pelagic species as previous studies in the Western English Channel have shown a very strong climatic influence that affects their abundance and distribution in the Channel [29], [34] and would therefore complicate the interpretation of the analysis. Landings reported as <1 t were omitted, as well as data collected prior to 1920 and between 1939–1945, since the older dataset excluded shellfish catches and there were data gaps during the World Wars.

Table 2 and 3 lists the 68 taxa analysed in our study and the respective TL obtained from FishBase [35] and the ‘Sea Around Us’ database [36]. For taxa reported at levels coarser than species, or under a general category (e.g. ‘sharks etc.’, ‘various shellfish’), a list of all marine species caught in UK waters was obtained from the Marine Management Organization (MMO) [37] and was used to derive an average trophic level for such categories. Tables S1 and S2 list the aggregated taxa and average trophic levels estimated for such groups. Species were also grouped into ISSCAAP categories (International Standard Statistical Classification of Aquatic Animals and Plants) to evaluate changes in landings composition over time. Table S3 shows taxa belonging to each ISSCAAP category.

**Table 2:** Finfish species included in our analysis with respective trophic levels (TL).

Common Name	Scientific Name	TL	Common Name	Scientific Name	TL
Witch	*Glyptocephalus cynoglossus*	3.1	Houndsharks, smoothhounds nei	Triakidae	3.9^a^
European flounder	*Platichthys flesus*	3.2	Red gurnard	*Chelidonichthys cuculus*	3.9
Lemon sole	*Microstomus kitt*	3.2	Small-eyed ray	*Raja microocellata*	3.9
Red mullet	*Mullus barbatus*	3.2	Blonde ray	*Raja brachyura*	4
Sand sole	*Pegusa lascaris*	3.2	Nursehound	*Scyliorhinus stellaris*	4
Common dab	*Limanda limanda*	3.3	Dogfish etc.	*Squalus* spp.	4.1^a^
Common sole	*Solea solea*	3.3	Dogfish sharks nei	Squalidae	4.1^a^
European plaice	*Pleuronectes platessa*	3.3	Haddock	*Melanogrammus aeglefinus*	4.1
Mullets nei	Mullidae	3.3^a^	Various sharks nei	Selachimorpha (Pleurotremata)	4.1^a^
Striped red mullet ( = Surmullet)	*Mullus surmuletus*	3.4	Blue shark	*Prionace glauca*	4.2
Grey gurnard	*Eutrigla gurnardus*	3.6	Megrim	*Lepidorhombus whiffiagonis*	4.2
Small-spotted catshark	*Scyliorhinus canicula*	3.6	Pollack	*Pollachius pollachius*	4.2
Pouting	*Trisopterus luscus*	3.7	Tope shark	*Galeorhinus galeus*	4.2
Saithe	*Pollachius virens*	3.7	European conger	*Conger conger*	4.3
Spotted ray	*Raja montagui*	3.7	Ling	*Molva molva*	4.3
Turbot	*Scophthalmus maximus*	3.7	Spurdog	*Squalus acanthias*	4.3
Brill	*Scophthalmus rhombus*	3.8	Atlantic cod	*Gadus morhua*	4.4
Groundfishes nei	Osteichthyes	3.8^a^	European Hake	*Merluccius merluccius*	4.4
Gurnards, searobins	*Triglidae*	3.8^a^	Whiting	*Merlangius merlangus*	4.4
Raja rays nei	*Raja* spp.	3.8^a^	Monkfish	*Lophius piscatorius*	4.5
Smooth-hound	*Mustelus mustelus*	3.8	Atlantic halibut	*Hippoglossus hippoglossus*	4.5
Thornback ray	*Raja clavata*	3.8	John dory	*Zeus faber*	4.5
Dogfishes and hounds nei	Squalidae, Scyliorhinidae	3.9^a^	Monkfish nei	*Lophius* spp.	4.5^a^

nei: not elsewhere included.

a Figure represent the mean TL value of known species belonging to that taxonomic group within UK waters. Species included were obtained from the UK Fisheries Statistics list [37], see Table S2.

**Table 3:** Invertebrate species included in our analysis with respective trophic levels (TL).

Common Name	Scientific Name	TL	Common Name	Scientific Name	TL
Blue mussel	*Mytilus edulis*	2	Velvet swimcrab	*Necora puber*	2.6
European flat oyster	*Ostrea edulis*	2	Common prawn	*Palaemon serratus*	2.7
Great Atlantic scallop	*Pecten maximus*	2	Marine crabs nei	Brachyura	2.8^a^
Pacific cupped oyster	*Crassostrea gigas*	2	Norway lobster	*Nephrops norvegicus*	2.9
Periwinkles nei	*Littorina* spp.	2^a^	Whelk	*Buccinum undatum*	3.1
Common edible cockle	*Cardium edule*	2.1	Common shrimp	*Crangon crangon*	3.2
Queen scallop	*Aequipecten opercularis*	2.1	Cuttlefish, bobtail squids nei	*Sepiidae, Sepiolidae*	3.5^a^
Spinous spider crab	*Maja squinado*	2.3	Common cuttlefish	*Sepia officinalis*	3.6
Variuos shellfish	Mollusca, Crustacea, Echinodermata	2.4^a^	Octopuses, etc. nei	Octopodidae	3.6^a^
Edible crab	*Cancer pagurus*	2.6	Various squids nei	Loliginidae, Ommastrephidae	4^a^
European lobster	*Homarus gammarus*	2.6	Common squids nei	*Loligo* spp.	4.2^a^

nei: not elsewhere included.

a Figure represent the mean TL value of known species belonging to that taxonomic group within UK waters. Species included were obtained from the UK Fisheries Statistics list [37], see Table S2.

## Results

**Figure 2:**
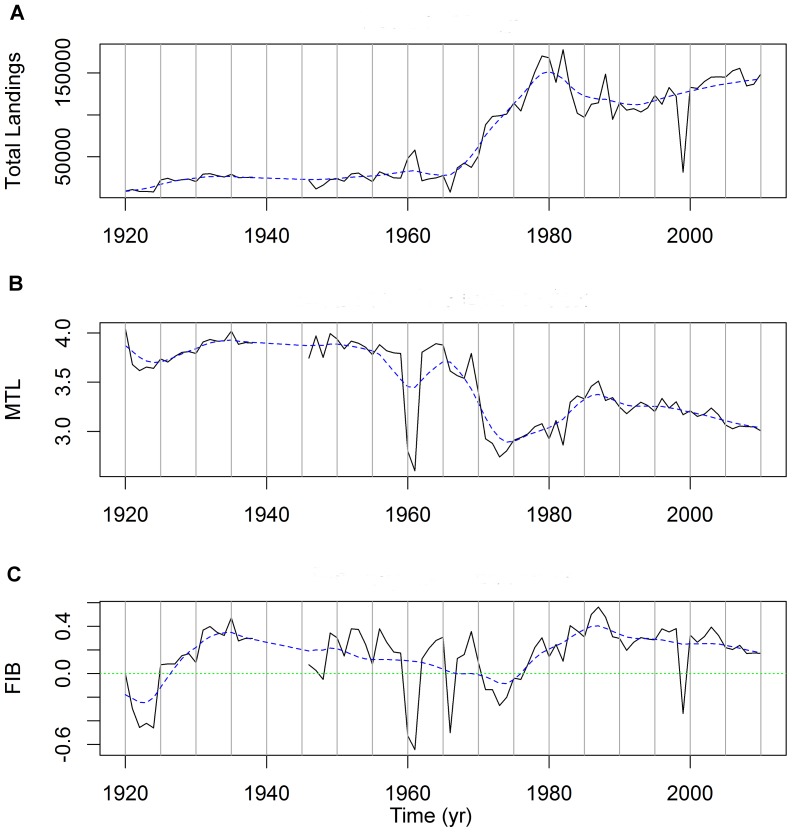
ICES data for the English Channel on landings, mTL and FiB index for 1920–2010. Analysis excludes pelagic species. (A) Annual landings from the English Channel. (B) Changes in the mTL over time. (C) Changes in the FiB index over time. The blue dashed line is a smoothing function, “supsmu” [50] available as standard with the R software package [51].

There was a clear increase in landings from the English Channel between 1920 and 2010 (Figure 2A). These increased gradually from 9,146 t in 1920 to 50,924 t in 1970 and escalated rapidly to a maximum weight of 177,793 t in 1982 t. These however fell abruptly to 96,783 t in 1985 and have stabilized at around 130,000–150,000 t in the last decade (Figure 2A).

The mTL of fish landed from the English Channel has declined from 4.0 in 1920 to 3.0 in 2010 (Figure 2B) and there is a marked negative correlation of −0.8 between mTL and total landings. Confirmatory statistical tests are difficult to support because of autocorrelation and non-linear trends. ‘Differencing’ the data is one way to reduce or remove the effects of autocorrelation, and when this was done the series were still negatively and significantly correlated with a coefficient of −0.30 (Pearson’s Product-Moment correlation, t = −2.7, df = 80, p = 0.0065). Between 1920 and 1970 both mTL and total landings underwent relatively little change but after the 1970s as catches increased mTL declined, the period of highest catches (1971–1982) corresponding to mTL values of 2.7–3.1. In the following years, catches declined considerably and mTL has continued to fall and was at 3.0 in 2010. An overall increase in the FiB index was also detected (Figure 2C), suggesting that the decline in mTL has indeed been compensated by increased catches as a consequence of either a geographic expansion of the fishery or increased productivity of the Channel.

We decided to use the concept of ‘Granger causality’ [38] to decide if there was any statistical evidence that mTL had ‘caused’ the change in FiB. We tested both whether mTL caused FiB and the reverse (whether FiB causes mTL) and the results are presented in Table 4. Clearly there is statistical evidence that changes in the mean trophic level ‘cause’ changes in the fishing balance index (p = 0.050) while, gratifyingly, the reverse is untrue (p = 0.315). For further statistical tests see Supporting Information S1.

**Table 4:** Granger causality testes at lag 1.

Model	Res. D.f.	D.f.	F-value	P(>F)
*‘FiB causes mTL’*				
FiB ∼ lags(fib) + lags(mTL)	80			
FiB ∼ lags(fib)	81	−1	1.024	0.315
*‘mTL causes FiB’*				
mTL ∼ lag(mTL) + lag(FiB)	80			
mTL ∼ lag(mTL)	81	−1	3.933	0.050

**Figure 3:**
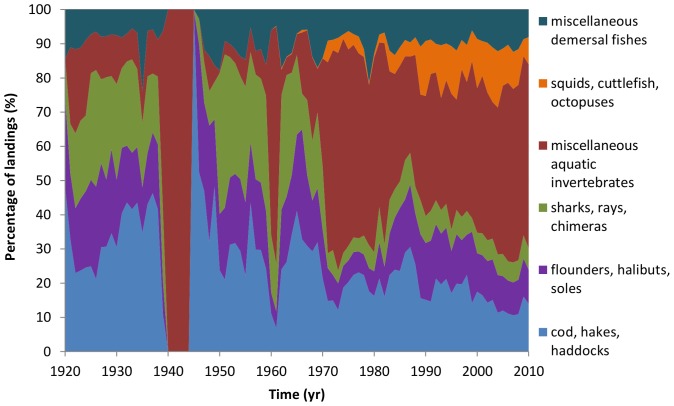
ICES data on changes in catch composition for the English Channel 1920–2010. Species grouped into ISSCAAP categories.

**Figure 4:**
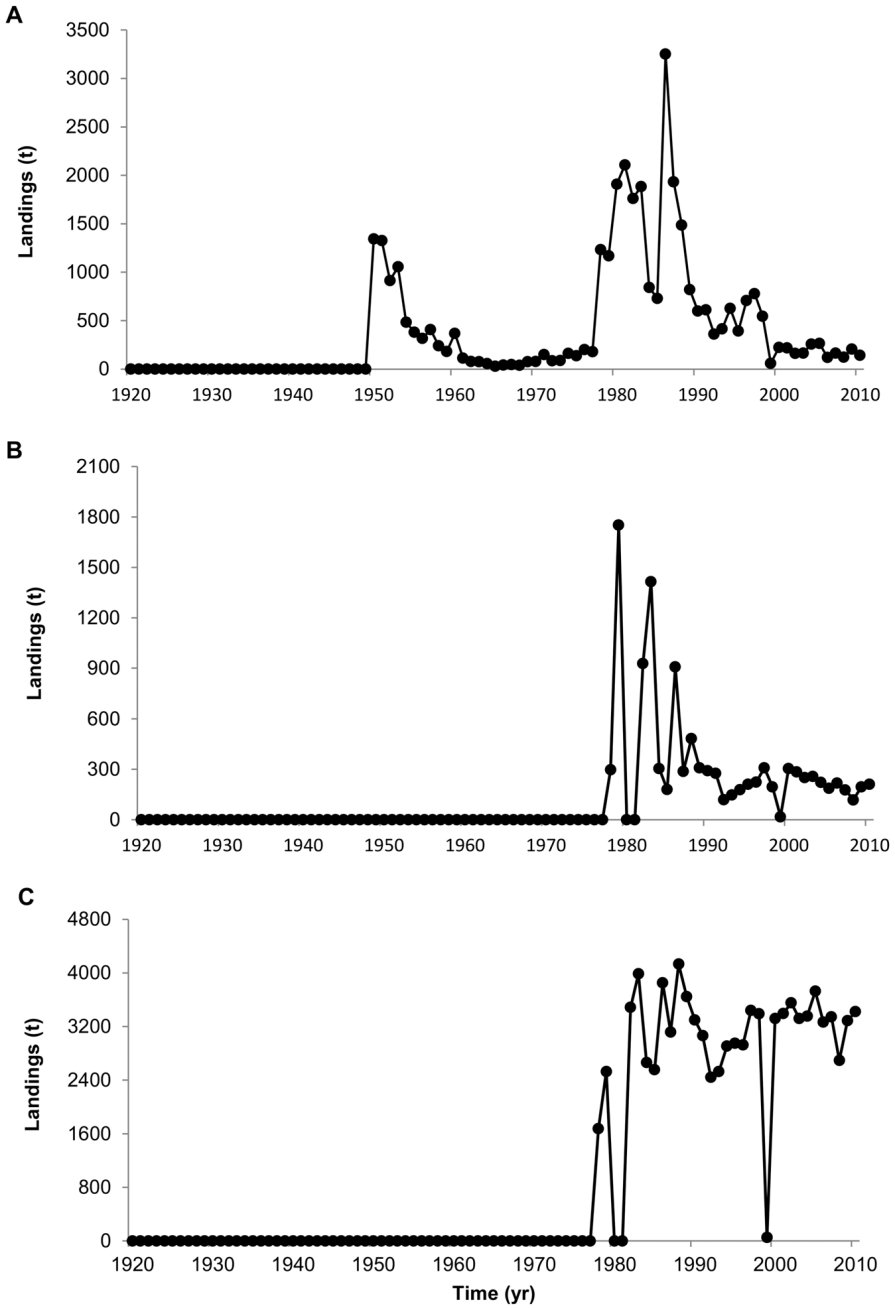
Annual landings of selected elasmobranch species. (A) Spurdog, (B) Tope shark, and (C) Small-spotted catshark.

**Figure 5:**
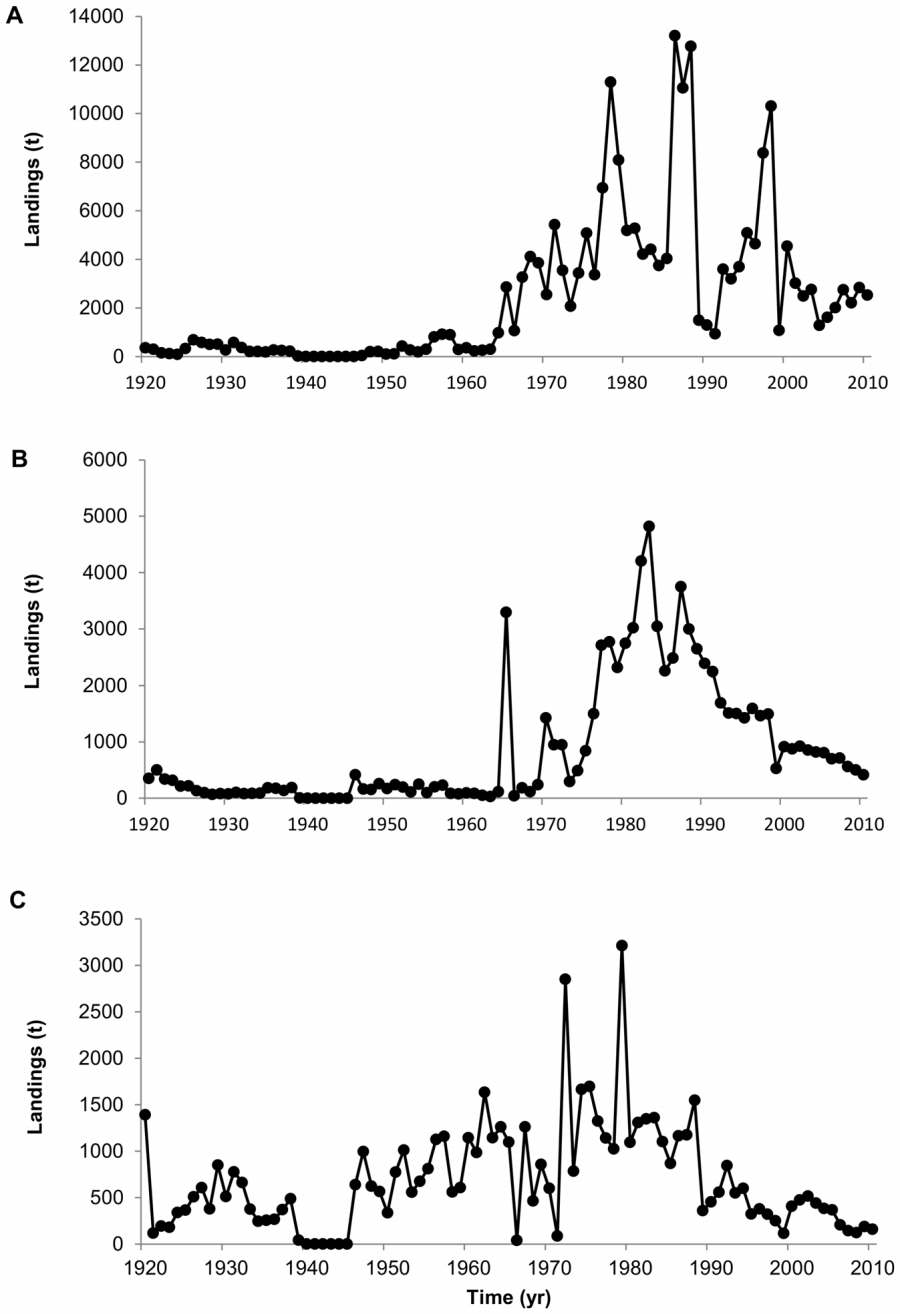
Annual landings of selected gadoid species. (A) Atlantic cod, (B) Ling, (C) European hake.

The composition of landings has also undergone changes over time with regards to both higher and lower trophic level species (Figure 3). The contribution of higher trophic level species to UK and France fisheries landings has decreased considerably in recent years. The group ‘sharks, rays, chimeras’ declined markedly from 34% in 1926 to 6.0% in 2010 with spurdog and tope shark (*Galeorhinus galeus*, Triakidae) landings declining considerably after the 1980s while small-spotted catshark landings increasing significantly (Figure 4). Similarly, the contribution of the ‘cods, haddock, hakes’ group has declined from 48% in 1920 to 14% in 2010. The most remarkable declines in landings have occurred for Atlantic cod (*Gadus morhua*, Gadidae), ling (*Molva molva*, Gadidae) and European hake (*Merluccius merluccius*, Merlucciidae) (Figure 5). Landings of ‘flounders, halibuts, soles’ and ‘miscellaneous demersal fishes’ has changed relatively little over the whole time-series (Figure 3).

**Figure 6:**
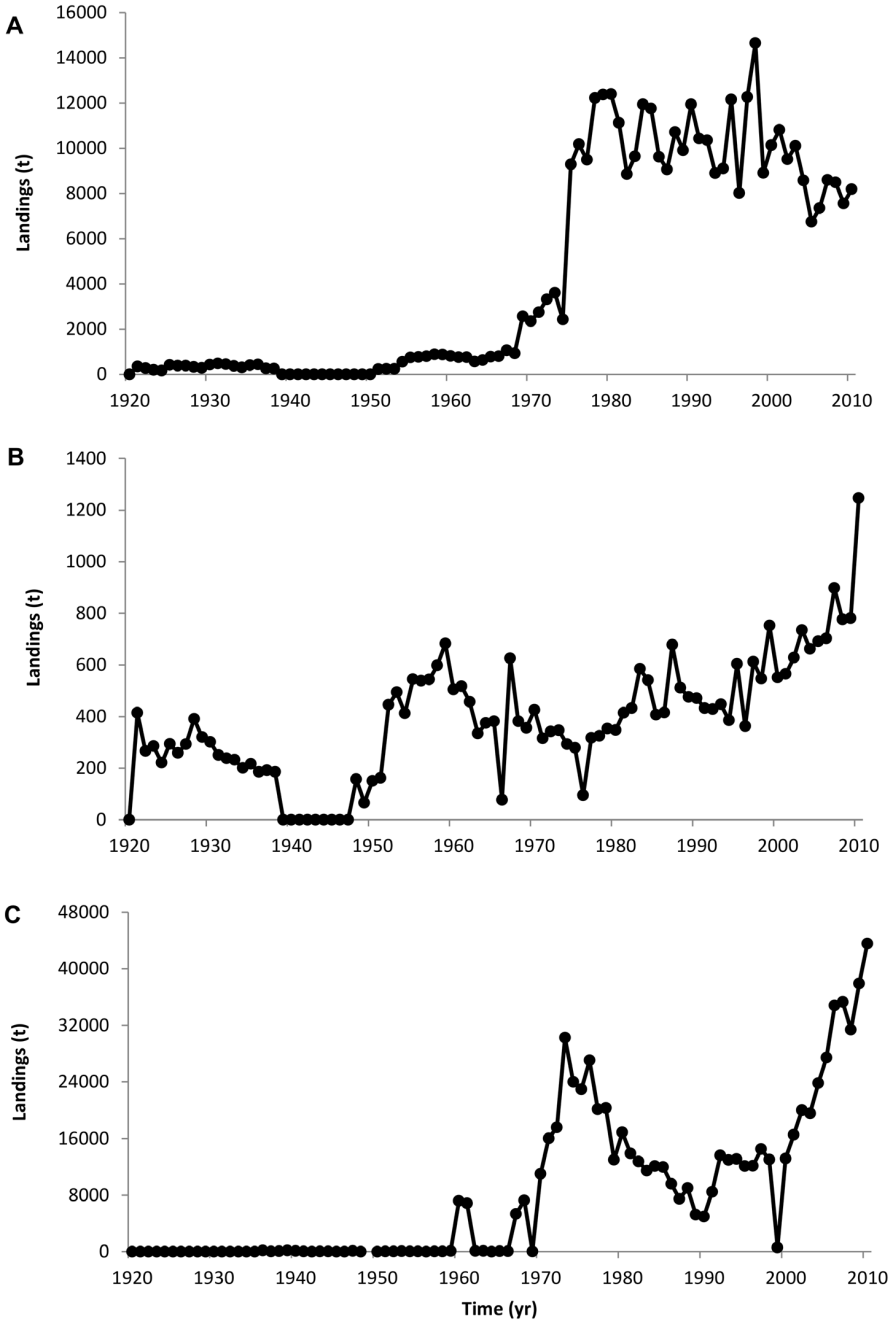
Annual landings of selected shellfish species. (A) Edible crab, (B) European lobster and (C) Great Atlantic scallop.

As for invertebrate species, a marked increase in landings from the English Channel is evident with the ‘miscellaneous aquatic invertebrates’ and ‘squids, cuttlefish, octopuses’ groups accounting to more than half of the total landings since the 1970s (Figure 3). In particular, landings of edible crab (*Cancer pagurus*, Cancridae), European lobster (*Homarus gammarus*, Nephropidae) and Great Atlantic scallop (*Pecten maximus*, Pectinidae) have increased considerably (Figure 6).

## Discussion

It is clear from our analyses that fishing pressure has caused significant changes to food webs of the English Channel over the past 90 years. The mean Trophic Level of English Channel landings has fallen by 0.1 unit per decade, one of the fastest rates reported among other heavily fished regions of the world providing yet more evidence that ‘fishing down food webs’ (*sensu* [5]) is a worldwide phenomenon (Table 1). The FiB index suggests that, either a geographic expansion of the Channel fishery or an increase in the productivity of the region has compensated for declining mTL with increased catches during two quite distinct periods in the time-series: 1925–1970 and 1980–2010. The former corresponds to the period of rapid industrialization and expansion of fishing documented by [28] and [39] for the English Channel and the UK respectively. Increases in landings were only maintained thanks to increased fishing efficiency and expansion into distant and deeper grounds, but stocks were declining long before the 1980s. Historical evidence reveals that signs of overfishing in UK waters were already apparent at the end of the 19^th^ century [40] and concerns regarding declining stocks date back at least to 1863 [3]. The second period matches an increase in water temperatures and overall productivity of the North East Atlantic during which stocks of cold-water species such as cod and haddock have seen a dramatic decline [41]. However, the declining trend of the FiB index in the last decade of the time series suggests that the factor that compensated for declining mTL in the second period is now fading away.

The landings data show a decrease in high trophic level species, such as gadoid fish and elasmobranchs, and an increase in low trophic level species such as invertebrates (Figure 3). The pattern is strikingly similar to that found elsewhere around the UK [11] and the rest of the world [10], [18], [22].

It is now well known that fishing removed populations of species that were common around Britain and Ireland a century ago. Large and long-lived species of elasmobranch, such as the common skate and the angel shark, have proved to be particularly vulnerable as they have low fecundity rates and are slow to mature [42], [43]. Conversely, small-spotted catshark have had a major increase in English Channel landings, this species has a very high rate of discard survival and matures earlier than larger species [28], [30]. Overall, English Channel elasmobranch landings have been declining steadily since the 1950s, with dramatic declines in spurdog, tope shark and thornback ray (*Raja clavata,* Rajidae).

When fishing pressure eased off during World Wars I and II stocks of demersal fish such as ‘cod, hake and haddock’ built up around the UK but recent decades of overfishing have brought these stocks to historic lows [39], [44]. The contribution of the ‘miscellaneous demersal’ group to total landings has changed little over time but landings of high trophic level species within this group, such as European conger (*Conger conger,* Congridae) and monk fish (*Lophius piscatorious,* Lophiidae), have declined since the 1960s as also documented in the Celtic Sea by [16].

The removal of high trophic level species can have a ‘cascading effect’ on lower trophic levels [1], [8]. In the English Channel, the proportion of lower trophic level species have increased over the years with invertebrate species accounting to more than 50% of the total landings since 1970s (Figure 3). The replacement of finfish species by invertebrates is a phenomenon which has been documented in many marine regions around the world and it has generally been followed by the development of new economically viable invertebrate fisheries [10], [11]. Despite their initial profitability, an increasing percentage of these new fisheries are already overexploited, collapsed or closed [12] and concerns have been raised where trawls and dredges are used to catch invertebrates as they degrade habitat complexity and species diversity [13].

Socioeconomic factors are also known to influence the composition of landings. These include consumers’ income and preferences, catching restrictions, fuel prices and technological innovations. These factors are in turn reflected in the price placed on a certain product. Generally as a resource become scarcer its price increases. In the Celtic Sea and in Portugal high trophic level species have become more expensive than low trophic level ones [16], [23] suggesting that the supply of those species has diminished but their demand is still high. Indeed the UK is a net importer of fish. 588,000 t of fish (excluding shellfish) were imported in 2010, with cod accounting for the highest import at 101,400 t [37]. Two-thirds of the total supply of haddock (60,300 t) is also derived from Iceland and Norway and in a lesser quantity also ling, hake and pollack are imported [37]. These are all fish species with catch restrictions in the UK but their landings have never fully met their allocated quotas. Conversely, in 2010 the UK exported 98,000 t of shellfish including 15,300 t of crab, 14,500 t of scallops and 16,500 t of shrimps and prawns [37]. However, [45] showed an increase in the price of low trophic level species globally in the past 50 years as a consequence of markets adjusting to the scarcity of target species by increasing the value of less desirable species, essentially creating ‘perverse incentives’ to unsustainable fishing. What is particularly compelling about the current study is that we’ve shown that by fishing or forcing down the food web, fishermen potentially create more biomass for themselves to harvest but there are many implications which remain to be investigated in detail of what seems a rather ‘perverse incentive’.

The recent recognition of a global fishery crisis has induced many governments and fishery scientists to question current strategies used in fisheries management. Since the 1950s, the concept of Maximum Sustainable Yield (MSY) and annual catch allowances has been at the foundation of all fisheries management [4]. However, disregard of scientific advices over the years has caused the deterioration of many fish stocks and has rendered the single-species approach highly ineffective [46]. As this study has demonstrated the impact of fisheries is much more pervasive than previously thought. Fisheries can affect the physical structure of an ecosystem as well as its ecological stability and productivity by depriving it of its components [1], [11]. The complexity of marine ecosystems compels a more holistic management approach that takes into account as many interactions as possible and whose priority is the integrity of the ecosystem from which the resource is extracted from [4].

There have been moves to improve the ecological status of the English Channel. In 2012, the UK Government approved 31 of 127 conservation areas recommended by an extensive stakeholder consultation, representing 3.7% of the marine area under English jurisdiction [47]. Removal of towed demersal fishing pressure from within such sites would allow habitat recovery and removal of all fishing effort within these areas could enhance adjacent fisheries through “spillover” or larval dispersal [48]. The Lyme Bay MPA in the English Channel is a great example of how an exploited ecosystem can regenerate and provide benefits to dependent fishing communities in only few years if closed to damaging fishing gear [49].

## Conclusion

The community-level changes observed in the English Channel reflect those that have occurred in other heavily-fished systems around Europe and the rest of the world [5], [10], [42]. The use of the Marine Trophic Index (MTI) and the Fisheries-in-Balance Index (FiB) on this long-term data series have helped expose a major shift from demersal fish to shellfish landings in the English Channel as a consequence of an unsustainable fishing practice fuelled by ‘perverse economic incentives’. These trends may be reversed by removing fishing pressure from within a network of closed areas and by implementing more rigid management measures including decommissioning schemes and reduction in fishing effort.

## Supporting Information

Table S1 **Estimated mean trophic level (TL) for aggregated taxa.**
(DOCX)

Table S2 **UK Fisheries Statistics list of species by group.**
(DOCX)

Table S3 **List of species arranged into ISCAAP groups.**
(DOCX)

Supporting Information S1 **Detrended mTL against Detrended FiB with statistical tests.**
(DOCX)
