# Supplementary material for: Overfishing and the Replacement of Demersal Finfish by Shellfish: An Example from the English Channel
Source: PLoS One. 2014 Jul 10;9(7):e101506. doi: 10.1371/journal.pone.0101506 (PMC4091961; doi:10.1371/journal.pone.0101506)
Supplement: Table S1 — Estimated mean trophic level (TL) for aggregated taxa. (DOCX) [file pone.0101506.s001.docx]

**Table S1.** **Estimated mean trophic level (TL) for aggregated taxa.**

| Group | Mean TL | Species | Binomial | TL |
| --- | --- | --- | --- | --- |
| Periwinkles nei | 2.0 |  |  |  |
|  |  | Common periwinkle | *Littorina littorea* | 2 |
| Various shellfish | 2.4 |  |  |  |
|  |  | Edible Crab | *Cancer pagurus* | 2.6 |
|  |  | Green Crab | *Carcinus maenas* | 3.5 |
|  |  | Spider Crabs | *Maja squinado* | 2.3 |
|  |  | Crabs - Velvet (Swim) | *Necora puber* | 2.6 |
|  |  | Common edible cockle | *Cardium edule* | 2.1 |
|  |  | Lobster - Squat | *Galatheidae* | 2.6 |
|  |  | European Lobster | *Homarus gammarus* | 2.6 |
|  |  | Mussels | *Mytilus edulis* | 2 |
|  |  | Norway Lobster | *Nephrops norvegicus* | 2.8 |
|  |  | Periwinkles nei | Littorina spp. | 2 |
|  |  | Native Oysters | *Ostrea edulis* | 2 |
|  |  | Pacific Oysters | *Crassostrea gigas* | 2 |
|  |  | Portuguese Oysters | Crassostrea spp | 2 |
|  |  | Great Atlantic Scallop | *Pecten maximus* | 2 |
|  |  | Queen Scallops | *Aequipecten opercularis* | 2.1 |
|  |  | Common Shrimp | *Crangon crangon* | 3.2 |
|  |  | Shrimps - Pink (Northern prawn) | *Pandalus borealis* | 2.5 |
|  |  | Pink Shrimps | *Pandalus montagui* | 2.3 |
|  |  | Common prawn | *Palaemon serratus* | 2.7 |
|  |  | Whelks | *Buccinum undatum* | 3.1 |
|  |  | Clams | *Mya arenaria* | 2 |
|  |  | Clams | *Mercenaria mercenaria* | 2 |
|  |  | Clams | *Venerupis decussata* | 2 |
|  |  | Crawfish | *Palinurus elephas* | 2.6 |
|  |  | European Razor Clam | *Solen vagina* | 2 |
|  |  | Green Sea Urchin | *Strongylocentrotus droebachiensis* | 2.3 |
|  |  | Surf Clams | *Spisula solida* | 2 |
| Marine crabs nei | 2.8 |  |  |  |
|  |  | Edible Crab | *Cancer pagurus* | 2.6 |
|  |  | Green Crab | *Carcinus maenas* | 3.5 |
|  |  | Spider Crabs | *Maja squinado* | 2.3 |
|  |  | Crabs - Velvet (Swim) | *Necora puber* | 2.6 |
| Mullets nei | 3.3 |  |  |  |
|  |  | Red mullet | *Mullus barbatus* | 3.2 |
|  |  | Striped red mullet(=Surmullet) | *Mullus surmuletus* | 3.4 |
| Cuttlefish,bobtail squids nei | 3.5 |  |  |  |
|  |  | Common cuttlefish | *Sepia officinalis* | 3.6 |
|  |  | Common bobtail | *Sepietta oweniana* | 3.5 |
|  |  | Atlantic bobtail | *Sepiola atlantica* | 3.5 |
|  |  |  | *Sepia elegans* | 3.5 |
| Octopuses, etc. nei | 3.6 |  |  |  |
|  |  | Common octopus | *Octopus vulgaris* | 3.5 |
|  |  | Curled Octopus | *Eledone cirrhosa* | 3.7 |
| Gurnards, searobins nei | 3.7 |  |  |  |
|  |  | Gurnards - Grey | *Eutrigla gurnardus* | 3.6 |
|  |  | Gurnards - Red | *Chelidonichthys cuculus* | 3.9 |
|  |  | Tub gurnard | *Chelidonichthys lucerna* | 3.7 |
| Groundfishes nei | 3.8 |  |  |  |
|  |  | Bass | *Dicentrarchus labrax* | 3.8 |
|  |  | Brill | *Scophthalmus rhombus* | 3.8 |
|  |  | Cod | *Gadus morhua* | 4.4 |
|  |  | European conger | *Conger conger* | 4 |
|  |  | Dabs | *Limanda limanda* | 3.3 |
|  |  | Long Rough Dabs | *Hippoglossoides platessoides* | 3.7 |
|  |  | Flounder | *Platichthys flesus* | 3.2 |
|  |  | Gurnards - Grey | *Eutrigla gurnardus* | 3.6 |
|  |  | Gurnards - Red | *Chelidonichthys cuculus* | 3.9 |
|  |  | Haddock | *Melanogrammus aeglefinus* | 4.1 |
|  |  | Hake | *Merluccius merluccius* | 4.4 |
|  |  | Halibut | *Hippoglossus hippoglossus* | 4.5 |
|  |  | Lemon Sole | *Microstomus kitt* | 3.2 |
|  |  | Ling | *Molva molva* | 4.3 |
|  |  | Megrim | *Lepidorhombus whiffiagonis* | 4.2 |
|  |  | Monk Fish | *Lophius piscatorius* | 4.5 |
|  |  | Plaice | *Pleuronectes platessa* | 3.3 |
|  |  | Pollack | *Pollachius pollachius* | 4.2 |
|  |  | Saithe | *Pollachius virens* | 3.7 |
|  |  | Sand Sole | *Solea lascaris* | 3.2 |
|  |  | Sole | *Solea solea* | 3.3 |
|  |  | Turbot | *Psetta maxima* | 3.7 |
|  |  | Whiting | *Merlangius merlangus* | 4.4 |
|  |  | Witch | *Glyptocephalus cynoglossus* | 3.1 |
|  |  | Black Scabbard Fish | *Aphanopus carbo* | 4.5 |
|  |  | Blue Antimora (Blue Hake) | *Antimora rostrata* | 3.6 |
|  |  | Blue Ling | *Molva dypterygia* | 4.5 |
|  |  | Bluemouth (Blue Mouth Redfish) | *Helicolenus dactylopterus* | 3.8 |
|  |  | Common Dragonet | *Callionymus lyra* | 3.3 |
|  |  | Common Mora | *Mora moro* | 3.8 |
|  |  | Deep-Water Cardinal Fish | *Epigonus telescopus* | 3.3 |
|  |  | Eelpout | *Zoarces viviparus* | 3.5 |
|  |  | Four-Spotted Megrim | *Lepidorhombus boscii* | 3.7 |
|  |  | Greater Weever | *Trachinus draco* | 4.2 |
|  |  | Greater Forked Beard | *Phycis blennoides* | 3.7 |
|  |  | John Dory | *Zeus faber* | 4.5 |
|  |  | Lumpfish | *Cyclopterus lumpus* | 3.9 |
|  |  | Pouting (Bib) | *Trisopterus luscus* | 3.7 |
|  |  | Red Mullet | *Mullus surmuletus* | 3.2 |
|  |  | Shore rockling | *Gaidropsarus mediterraneus* | 3.4 |
|  |  | Three-bearded rockling | *Gaidropsarus vulgaris* | 3.3 |
|  |  | Red Scorpionfish | *Scorpaena scrofa* | 4.2 |
|  |  | Wreckfish | *Polyprion americanus* | 4.1 |
| Raja rays nei | 3.8 |  |  |  |
|  |  | Blonde Ray | *Raja brachyura* | 4 |
|  |  | Common Skate(Blue/Grey) | *Dipturus batis (previously Raja)* | 4 |
|  |  | Cuckoo Ray | *Leucoraja naevus (previously Raja)* | 3.9 |
|  |  | Long-nosed Skate | *Dipturus oxyrinchus (previously Raja)* | 3.5 |
|  |  | Sandy Ray | *Leucoraja circularis (previously Raja)* | 3.5 |
|  |  | Skate (Round) | *Raja fyllae* | 3.8 |
|  |  | Small-eyed Ray | *Raja microocellata* | 3.9 |
|  |  | Spotted Ray | *Raja montagui* | 3.7 |
|  |  | Shagreen Ray | *Leucoraja fullonica (previously Raja)* | 3.5 |
|  |  | Starry Ray | *Amblyraja radiata (previously Raja)* | 4 |
|  |  | Thornback Ray | *Raja clavata* | 3.8 |
|  |  | Undulate Ray | *Raja undulata* | 3.5 |
|  |  | White Skate | *Rostroraja alba* | 4.4 |
| Houndsharks,smoothhounds nei | 3.9 |  |  |  |
|  |  | Starry Smooth Hound | *Mustelus asterias* | 3.7 |
|  |  | Smooth-hound | *Mustelus mustelus* | 3.8 |
|  |  | Tope | *Galeorhinus galeus* | 4.2 |
| Dogfishes and hounds nei | 3.9 |  |  |  |
|  |  | Starry Smooth Hound | *Mustelus asterias* | 3.7 |
|  |  | Smooth-hound | *Mustelus mustelus* | 3.8 |
|  |  | Tope | *Galeorhinus galeus* | 4.2 |
|  |  | Lesser Spotted Dog | *Scyliorhinus canicula* | 3.6 |
|  |  | Spurdog (Piked dogfish) | *Squalus acanthias* | 4.3 |
|  |  | Nursehound | *Scyliorhinus stellaris* | 4 |
| Various squids nei | 4.0 |  |  |  |
|  |  | European squid | *Loligo vulgaris* | 4.1 |
|  |  | European flying squid | *Todarodes sagittatus* | 4 |
|  |  | Northern shortfin squid | *Illex illecebrosus* | 3.9 |
|  |  | Broadtail shortfin squid | *Illex coindetii* | 4.1 |
|  |  | Veined Squid | *Loligo forbesii* | 4.3 |
|  |  | European common squid | *Alloteuthis subulata* | 3.8 |
|  |  |  |  |  |
| Various sharks nei | 4.1 |  |  |  |
|  |  | Birdbeak Dogfish | *Deania calcea* | 4.2 |
|  |  | Black Dogfish | *Centroscyllium fabricii* | 3.9 |
|  |  | Blackmouthed Dogfish | *Galeus melastomus* | 4.2 |
|  |  | Lesser Spotted Dog | *Scyliorhinus canicula* | 3.6 |
|  |  | Spurdog (Piked dogfish) | *Squalus acanthias* | 4.3 |
|  |  | Nursehound | *Scyliorhinus stellaris* | 4 |
|  |  | Smooth-hound | *Mustelus mustelus* | 3.8 |
|  |  | Angel Shark | *Squatina squatina* | 4.1 |
|  |  | Blue Shark | *Prionace glauca* | 4.2 |
|  |  | Frilled Shark | *Chlamydoselachus anguineus* | 4.2 |
|  |  | Great Lanternshark | *Etmopterus princeps* | 4.2 |
|  |  | Greenland Shark | *Somniosus microcephalus* | 4.2 |
|  |  | Gulper Shark | *Centrophorus granulosus* | 4.1 |
|  |  | Kitefin Shark | *Dalatias licha* | 4.2 |
|  |  | Porbeagle | *Lamna nasus* | 4.5 |
|  |  | Six-Gilled Shark | *Hexanchus griseus* | 4.3 |
|  |  | Smooth Hammerhead | *Sphyrna Zygaena* | 4.5 |
|  |  | Starry Smooth Hound | *Mustelus asterias* | 3.7 |
|  |  | Thresher Shark | *Alopias vulpinus* | 4.5 |
|  |  | Tope | *Galeorhinus galeus* | 4.2 |
| Dogfish sharks nei | 4.1 |  |  |  |
|  |  | Birdbeak Dogfish | *Deania calcea* | 4.2 |
|  |  | Black Dogfish | *Centroscyllium fabricii* | 3.9 |
|  |  | Spurdog (Piked dogfish) | *Squalus acanthias* | 4.3 |
| Dogfish etc | 4.1 |  |  |  |
|  |  | Birdbeak Dogfish | *Deania calcea* | 4.2 |
|  |  | Black Dogfish | *Centroscyllium fabricii* | 3.9 |
|  |  | Spurdog (Piked dogfish) | *Squalus acanthias* | 4.3 |
| Common squids nei | 4.2 |  |  |  |
|  |  | European squid | *Loligo vulgaris* | 4.1 |
|  |  | Veined Squid | *Loligo forbesii* | 4.3 |
|  |  |  |  |  |
| Monkfish nei | 4.5 |  |  |  |
|  |  | Monk Fish | *Lophius piscatorius* | 4.5 |
|  |  | Blackbellied angler | *Lophius budegassa* | 4.5 |

nei: not elsewhere included
