# Supplementary material for: Overfishing and the Replacement of Demersal Finfish by Shellfish: An Example from the English Channel
Source: PLoS One. 2014 Jul 10;9(7):e101506. doi: 10.1371/journal.pone.0101506 (PMC4091961; doi:10.1371/journal.pone.0101506)
Supplement: Table S2 — UK Fisheries Statistics list of species by group. (DOCX) [file pone.0101506.s002.docx]

**Table S2**. **UK Fisheries Statistics list of species by group**.

| Code | UKSFS species | Including: | Latin Name |
| --- | --- | --- | --- |
| Demersal |  |  |  |
| BSS | Bass |  | *Dicentrarchus labrax* |
| BLL | Brill |  | *Scophthalmus rhombus* |
|  | Catfish |  |  |
| CAA |  | Catfish | *Anarhichas lupus* |
| CAT |  | Catfish | Anarhichas spp |
| CAX |  | Sea Catfishes | Ariidae |
| COD | Cod |  | *Gadus morhua* |
| COE | Conger Eels |  | *Conger conger* |
|  | Dabs |  |  |
| DAB |  | Dabs | *Limanda limanda* |
| PLA |  | Long Rough Dabs | *Hippoglossoides platessoides* |
|  | Dogfish |  |  |
| DCA |  | Birdbeak Dogfish | *Deania calcea* |
| CFB |  | Black Dogfish | *Centroscyllium fabricii* |
| SHO |  | Blackmouthed Dogfish | *Galeus melastomus* |
| SYX |  | Dogfish (Scyliorhinidae) | *Scyliorhinidae* |
| SYR |  | Knifetooth Dogfish | *Scymnodon ringens* |
| SYC |  | Lesser Spotted Dog | *Scyliorhinus canicula* |
| CYP |  | Longnose Velvet Dogfish | *Centroscymnus crepidater* |
| DGS |  | Spurdog | *Squalus acanthias* |
| DGH |  | Unidentified Dogfish | Squalidae, Scyliorhinidae |
| FLE | Flounder |  | *Platichthys flesus* |
|  | Gurnard |  |  |
| GUX |  | Gurnard and Latchet | Triglidae |
| GUG |  | Gurnards - Grey | *Eutrigla gurnardus* |
| GUR |  | Gurnards - Red | *Chelidonichthys cuculus* |
| HAD | Haddock |  | *Melanogrammus aeglefinus* |
| HKE | Hake |  | *Merluccius merluccius* |
| HAL | Halibut |  | *Hippoglossus hippoglossus* |
| GHL | Halibut, Greenland |  | *Reinhardtius hippoglossoides* |
| LEM | Lemon Sole |  | *Microstomus kitt* |
| LIN | Ling |  | *Molva molva* |
| LEZ | Megrim |  | Lepidorhombus spp |
| ANF | Monks or Anglers |  | Lophiidae |
| PLE | Plaice |  | *Pleuronectes platessa* |
| POL | Pollack (Lythe) |  | *Pollachius pollachius* |
| RED | Redfish |  | Sebastes spp |
| POK | Saithe |  | *Pollachius virens* |
| SAN | Sand Eels |  | Ammodytes spp |
|  | Skates and Rays |  |  |
| RJG |  | Arctic Skate | *Raja hyperborea* |
| RJH |  | Blonde Ray | *Raja brachyura* |
| RJB |  | Common Skate(Blue/Grey) | *Dipturus batis (previously Raja)* |
| JDP |  | Common Stingray | *Dasyatis pastinaca* |
| RJN |  | Cuckoo Ray | *Leucoraja naevus (previously Raja)* |
| TTO |  | Electric Ray | *Torpedo nobiliana* |
| RJO |  | Long-nosed Skate | *Dipturus oxyrinchus (previously Raja)* |
| TTR |  | Marbled Electric Ray | *Torpedo marmorata* |
| JAD |  | Norwegian Skate | *Dipturus nidarosiensis (previously Raja)* |
| RJY |  | Skate (Round) | *Raja fyllae* |
| RJI |  | Sandy Ray | *Leucoraja circularis (previously Raja)* |
| RJF |  | Shagreen Ray | *Leucoraja fullonica (previously Raja)* |
| SKA |  | Skates and Rays | Raja spp |
| RJE |  | Small-eyed Ray | *Raja microocellata* |
| RJM |  | Spotted Ray | *Raja montagui* |
| RJR |  | Starry Ray | *Amblyraja radiata (previously Raja)* |
| RJC |  | Thornback Ray | *Raja clavata* |
| RJU |  | Undulate Ray | *Raja undulata* |
| RJA |  | White Skate | *Rostroraja alba (Raja Alba in FAO)* |
|  | Sole |  |  |
| SOS |  | Sand Sole | *Solea lascaris* |
| SOL |  | Sole | *Solea solea* |
| USK | Torsk (Tusk) |  | *Brosme brosme* |
| TUR | Turbot |  | *Psetta maxima* |
| WHG | Whiting |  | *Merlangius merlangus* |
| WIT | Witch |  | *Glyptocephalus cynoglossus* |
| AGN |  | Angel Shark | *Squatina squatina* |
| HKP |  | Argentine Hake (Sw Atlantic) | *Merluccius hubbsi* |
| SBA |  | Axillary Seabream | *Pagellus acarne* |
| ALC |  | Baird's Smoothhead | *Alepocephalus bairdii* |
| USB |  | Ballan Wrasse | *Labrus bergylta* |
| BSK |  | Basking Shark | *Cetorhinus maximus* |
| ALF |  | Beryx | Beryx spp |
| BSF |  | Black Scabbard Fish | *Aphanopus carbo* |
| BRB |  | Black Seabream | *Spondyliosoma cantharus* |
| ANT |  | Blue Antimora (Blue Hake) | *Antimora rostrata* |
| BLI |  | Blue Ling | *Molva dypterygia* |
| BSH |  | Blue Shark | *Prionace glauca* |
| BLU |  | Bluefish | *Pomatomus Saltatrix* |
| BRF |  | Bluemouth (Blue Mouth Redfish) | *Helicolenus dactylopterus* |
| BOC |  | Boarfish (Capros Aper) | *Capros aper* |
| LFO |  | Brain Root Coral | *NULL* |
| POA |  | Bream - Ray's | *Brama brama* |
| CAA |  | Catfish | *Anarhichas lupus* |
| CAT |  | Catfish | Anarhichas spp |
| LYY |  | Common Dragonet | *Callionymus lyra* |
| RIB |  | Common Mora | *Mora moro* |
| COE |  | Conger Eels | *Conger conger* |
| DAB |  | Dabs | *Limanda limanda* |
| IOO |  | Dark Flounder | *Pseudipleuronectes Obscurus* |
| EPI |  | Deep-Water Cardinal Fish | *Epigonus telescopus* |
| REB |  | Deep-Water Redfish (Rose Fish) | *Sebastes mentella* |
| GPD |  | Dusky Perch (Grouper) | *Epinephelus marginatus* |
| ELP |  | Eelpout | *Zoarces viviparus* |
| ELE |  | Eels | *Anguilla anguilla* |
| PGO |  | Flatnose Catfish | *Anaspidoglanis Macrostoma* |
| FLE |  | Flounder or Flukes | *Platichthys flesus* |
| FOR |  | Forkbeard | *Phycis phycis* |
| LDB |  | Four-Spotted Megrim | *Lepidorhombus boscii* |
| HXC |  | Frilled Shark | *Chlamydoselachus anguineus* |
| SBG |  | Gilt-Head Seabream | *Sparus aurata* |
| BYS |  | Golden Eye Perch | *Beryx splendens* |
| ETR |  | Great Lanternshark | *Etmopterus princeps* |
| GFB |  | Greater Forked Beard | *Phycis blennoides* |
| WEG |  | Greater Weever | *Trachinus draco* |
| GSK |  | Greenland Shark | *Somniosus microcephalus* |
| GRX |  | Grunts | *Haemulidae (=Pomadasyidae)* |
| GUP |  | Gulper Shark | *Centrophorus granulosus* |
| GHL |  | Halibut - Greenland | *Reinhardtius hippoglossoides* |
| SPN |  | Hammerheads | Sphyrna spp |
| GRN |  | Hoki-Macruronus Novazelandiae | *Macruronus novaezelandiae* |
| APQ |  | Iceland Catshark | *Apristurus laurussonii* |
| JOD |  | John Dory | *Zeus faber* |
| KCP |  | Kingklip | *Genypterus capensis* |
| SCK |  | Kitefin Shark | *Dalatias licha* |
| DEL |  | Large-Eyed Dentex | *Dentex macrophthalmus* |
| CYH |  | Large-Eyed Rabbit Fish | *Hydrolagus mirabilis* |
| GUQ |  | Leafscale Gulper Shark | *Centrophorus squamosus* |
| LVR |  | Livers | *NULL* |
| PLA |  | Long Rough Dabs | *Hippoglossoides platessoides* |
| ANK |  | Lophius Budegassa (BSDB) | *Lophius budegassa* |
| LUM |  | Lumpfish | *Cyclopterus lumpus* |
| MAK |  | Mako Shark | Isurus spp |
| GAM |  | Mouse Catshark | *Galeus murinus* |
| MUL |  | Mullet - Other | Mugilidae |
| NEC |  | New Zealand Red Cod | *Pseudophycis bachus* |
| NOP |  | Norway Pout | *Trisopterus esmarkii* |
| OIL |  | Oilfish | *Ruvettus Pretiosus* |
| ORY |  | Orange Roughy (Emperor Fish) | *Hoplostethus atlanticus* |
| FLX |  | Other Flatfish | *Pleuronectiformes* |
| GRO |  | Other or mixed Demersal | Osteichthyes |
| BLP |  | Patagonian Blennie | *Eleginops maclovinus* |
| TOP |  | Patagonian Toothfish | *Dissostichus eleginoides* |
| POR |  | Porbeagle | *Lamna nasus* |
| CYO |  | Portuguese Dogfish (Shark) | *Centroscymnus coelolepis* |
| BIB |  | Pouting (Bib) | *Trisopterus luscus* |
| EEO |  | Queen Snapper | *Etelis oculatus* |
| CMO |  | Rabbit Fish (Rattail) | *Chimaera monstrosa* |
| SBR |  | Red (Blackspot) Seabream | *Pagellus bogaraveo* |
| CBC |  | Red Bandfish | *Cepola rubescens* |
| MUR |  | Red Mullet | *Mullus surmuletus* |
| RSE |  | Red Scorpionfish | *Scorpaena scrofa* |
| RED |  | Redfishes | Sebastes spp |
| PHO |  | Risso S Smoothhead | *Alepocephalus rostratus* |
| ROL |  | Rockling | Gaidropsarus spp |
| ROE |  | Roes | *NULL* |
| RHG |  | Roughead Grenadier | *Macrourus berglax* |
| RNG |  | Roundnose Grenadier | *Coryphaenoides rupestris* |
| OXN |  | Sailfin Roughshark (Sharpback) | *Oxynotus paradoxus* |
| SAL |  | Salmon | *Salmo salar* |
| ATP |  | Sand Smelt | *Atherina presbyter* |
| SPL |  | Scalloped Hammerhead Shark | *Sphyrma Lewini* |
| SBX |  | Sea Breams | Sparidae |
| CAX |  | Sea Catfishes | Ariidae |
| TRS |  | Sea Trout | *Salmo trutta* |
| SKH |  | Sharks | *Selachimorpha(*Pleurotremata*)* |
| FAL |  | Silky Shark | *Carcharhinus falciformis* |
| HPR |  | Silver Roughy (Pink) | *Hoplostethus mediterraneus* |
| SFS |  | Silver Scabbard Fish | *Lepidopus caudatus* |
| SBL |  | Six-Gilled Shark | *Hexanchus griseus* |
| SPZ |  | Smooth Hammerhead | *Sphyrna Zygaena* |
| SMD |  | Smoothhound | *Mustelus mustelus* |
| LEC |  | Snake Mackerel | *Escolar Lepidocybium* |
| TJX |  | Spiny Scorpionfish | *Trachyscorpia cristulata* |
| SDS |  | Starry Smooth Hound | *Mustelus asterias* |
| RCT |  | Straightnose Rabbitfish | *Rhinochimaera atlantica* |
| STU |  | Sturgeon | Acipenseridae |
| FIL |  | Threadsail Filefish | *Stephanolepis cirrhifer* |
| THR |  | Thresher Shark | Alopias spp |
| GAG |  | Tope | *Galeorhinus galeus* |
| USK |  | Torsk (Tusk) | *Brosme brosme* |
| TRI |  | Triggerfish | Balistidae |
| DGX |  | Unid DS Squal Sharks & Dogfish | Squalidae |
| ETX |  | Velvet Belly | *Etmopterus spinax* |
| CET |  | Wedge Sole | *Dicologlossa cuneata* |
| WRA |  | Wrasses | Labridae |
| WRF |  | Wreckfish | *Polyprion americanus* |
| YEL |  | Yellowtail Flounder | *Limanda ferruginea* |
|  |  |  |  |
| Pelagic |  |  |  |
| WHB | Blue Whiting |  | *Micromesistius poutassou* |
| HER | Herring |  | *Clupea harengus* |
| JAX | Horse Mackerel |  | Trachurus spp |
| MAC | Mackerel |  | *Scomber scombrus* |
|  | Sardines |  |  |
| SAA |  | Sardinelle Aurita (Round) | *Sardinella aurita* |
| SAE |  | Sardinelle Maderensis (Flat) | *Sardinella maderensis* |
| PIL |  | Pilchards | *Sardina pilchardus* |
| SPR | Sprats |  | *Sprattus sprattus* |
|  | Tuna |  |  |
| ALB |  | Albacore | *Thunnus alalunga* |
| BET |  | Bigeye Tuna | *Thunnus obesus* |
| BFT |  | Blue Fin Tuna | *Thunnus thynnus* |
| BON |  | Bonito | *Sarda sarda* |
| FRZ |  | Frigate and bullet tunas | *Auix thazard A rochei* |
| LTA |  | Little Tuna | *Euthynnus Alletteratus* |
| YFT |  | Yellowfin tuna | *Thunnus albacares* |
| SBF |  | Southern Blue Fin Tuna | *Thunnus Maccoyii* |
| TUX |  | Tuna - Other | Scombroidei |
| ALB |  | Albacore | *Thunnus alalunga* |
| ANE |  | Anchovy | *Engraulis encrasicolus* |
| BTH |  | Big-eye thresher | *Alopias superciliosus* |
| BET |  | Bigeye Tuna | *Thunnus obesus* |
| BLM |  | Black Marlin | *Makaira indica* |
| BFT |  | Blue Fin Tuna | *Thunnus thynnus* |
| BUM |  | Blue Marlin | *Makaira nigricans* |
| BOG |  | Bogues | *Boops boops* |
| BON |  | Bonito | *Sarda sarda* |
| CAP |  | Capelin | *Mallotus villosus* |
| DCO |  | Common Dolphin | *Delphinus delphis* |
| DOL |  | Common dolphinfish | *Coryphaena hippurus* |
| FRZ |  | Frigate and bullet tunas | *Auix thazard A rochei* |
| GAR |  | Garfish | *Belone belone* |
| ARU |  | Greater Silver Smelt | *Argentina silus* |
| SFA |  | Indo-Pacific Sailfish | *Istiophorus platypterus* |
| LEE |  | Leerfish | *Lichia Amia* |
| LTA |  | Little Tuna | *Euthynnus Alletteratus* |
| LMA |  | Longfin mako | *Isurus paucus* |
| MGR |  | Meagre Fish | *Argyrosomus regius* |
| PEL |  | Other Pelagic | Osteichthyes |
| PTH |  | Pelagic thresher | *Alopias pelagicus* |
| POX |  | Pompanos Nei | Trachinotus Spp |
| BIL |  | Sailfish | *Istiophoridae* |
| COL |  | Sardinia Coral | *Corallium Rubrum* |
| SHD |  | Shad | *Alosa alosa, A.fallax* |
| SMA |  | Shortfin mako | *Isurus oxyrinchus* |
| SME |  | Smelt (European) | *Osmerus eperlanus* |
| SBF |  | Southern Blue Fin Tuna | *Thunnus Maccoyii* |
| SPR |  | Sprats | *Sprattus sprattus* |
| MOP |  | Sunfish | Mola spp |
| SWO |  | Swordfish | *Xiphias gladius* |
| TUX |  | Tuna - Other | Scombroidei |
| SPV |  | Whitefin hammerhead | *Sphyrna couardi* |
| YFT |  | Yellowfin tuna | *Thunnus albacares* |
| YTC |  | Yellowtail Amberjack | *Seriola lalandi* |
|  |  |  |  |
| Shellfish |  |  |  |
| COC | Cockles |  | *Cerastoderma edule* |
|  | Crabs |  |  |
| CRE |  | Crabs (C.P.Mixed Sexes) | *Cancer pagurus* |
| CRR |  | Deepwater Red Crab | *Geryon quinquedens* |
| CRG |  | Green Crab | *Carcinus maenas* |
| KCS |  | King Crab | Paralithodes spp |
| CRA |  | Mixed Crabs | Brachyura |
| SCR |  | Spider Crabs | *Maja squinado* |
| KCX |  | Stone Crab | Lithodidae |
| LIO |  | Crabs - Velvet (Swim) | *Necora puber* |
| CTL | Cuttlefish |  | *Sepiidae, Sepiolidae* |
|  | Lobster |  |  |
| LOQ |  | Lobster - Squat | Galatheidae |
| LBE |  | Lobsters | *Homarus gammarus* |
| MUS | Mussels |  | *Mytilus edulis* |
| NEP | Nephrops |  | *Nephrops norvegicus* |
| PER | Periwinkles |  | Littorina spp |
|  | Oysters |  |  |
| OYX |  | Oysters | Ostrea spp |
| OYF |  | Native Oysters | *Ostrea edulis* |
| OYG |  | Pacific Oysters | *Crassostrea gigas* |
| OYC |  | Portuguese Oysters | Crassostrea spp |
|  | Scallops |  |  |
| SCE |  | Scallops | *Pecten maximus* |
| QSC |  | Queen Scallops | *Aequipecten opercularis* |
|  | Shrimps and Prawns |  |  |
| CSH |  | Brown Shrimps | *Crangon crangon* |
| PRA |  | Shrimps - Pink (Northern prawn) | *Pandalus borealis* |
| PAN |  | Pink Shrimps | Pandalus spp |
| PEN |  | Shrimps - Other | Penaeus spp |
|  | Squid |  |  |
| SQA |  | Argentine shortfin squid | *Illex argentinus* |
| SQE |  | European Flying Squid | *Todarodes sagittatus* |
| SQU |  | Mixed Squid and Octopi | Loliginidae, Ommastrephidae |
| SQP |  | Patagonian squid | *Loligo gahi* |
| SQC |  | Squid | Loligo spp |
| WHE | Whelks |  | *Buccinum undatum* |
|  | Other Shellfish |  |  |
| STF |  | Asteroidea Nei. | Asteroidea |
| TGS |  | Caramote Prawn | *Penaeus kerathurus* |
| CLS |  | Clams (M.Arenaria) | *Mya arenaria* |
| CLH |  | Clams (M.Mercenaria) | *Mercenaria mercenaria* |
| CLV |  | Clams (V.Decussata) | Veneridae |
| CPR |  | Common Prawns | *Palaemon serratus* |
| CRW |  | Crawfish | Palinurus spp |
| CMM |  | Manilla Clam | *Corbicula manilensis* |
| CLX |  | Mixed Clams | Bivalvia |
| OYF |  | Native Oysters | *Ostrea edulis* |
| OCT |  | Octopus | Octopodidae |
| CRU |  | Other Crustaceans | Crustacea |
| MOL |  | Other Molluscs | Mollusca |
| ZZA |  | Other Shellfish | *NULL* |
| OYX |  | Oysters | Ostrea spp |
| OYG |  | Pacific Oysters | *Crassostrea gigas* |
| PER |  | Periwinkles | Littorina spp |
| OYC |  | Portuguese Oysters | Crassostrea spp |
| RAZ |  | Razor Clam | Solen spp |
| URC |  | Sea Urchin | Strongylocentrotus spp |
| ILL |  | Shortfin squids | Illex spp |
| SSD |  | Surf Clams | Spisula spp |
