# Supplementary material for: Overfishing and the Replacement of Demersal Finfish by Shellfish: An Example from the English Channel
Source: PLoS One. 2014 Jul 10;9(7):e101506. doi: 10.1371/journal.pone.0101506 (PMC4091961; doi:10.1371/journal.pone.0101506)
Supplement: Table S3 — List of species arranged into ISCAAP groups. (DOCX) [file pone.0101506.s003.docx]

**Table S3**. **List of species arranged into ISCAAP groups.**

| ISSCAAP group | Common Name | Scientific Name |
| --- | --- | --- |
| Cod, hakes, haddocks | Atlantic cod | *Gadus morhua* |
|  | European hake | *Merluccius merluccius* |
|  | Haddock | *Melanogrammus aeglefinus* |
|  | Ling | *Molva molva* |
|  | Pollack | *Pollachius pollachius* |
|  | Saithe | *Pollachius virens* |
|  | Pouting (=Bib) | *Trisopterus luscus* |
|  | Whiting | *Merlangius merlangus* |
| Flounders, halibuts, soles | Atlantic halibut | *Hippoglossus hippoglossus* |
|  | Common dab | *Limanda limanda* |
|  | Common sole | *Solea solea* |
|  | European flounder | *Platichthys flesus* |
|  | European plaice | *Pleuronectes platessa* |
|  | Lemon sole | *Microstomus kitt* |
|  | Sand sole | *Pegusa lascaris* |
|  | Turbot | *Scophthalmus maximus* |
|  | Megrim | *Lepidorhombus whiffiagonis* |
|  | Brill | *Scophthalmus rhombus* |
|  | Witch flounder | *Glyptocephalus cynoglossus* |
| Sharks, rays, chimaeras | Various sharks nei | *Selachimorpha (*Pleurotremata*)* |
|  | Tope shark | *Galeorhinus galeus* |
|  | Blue shark | *Prionace glauca* |
|  | Dogfish etc | Squalus spp. |
|  | Dogfish sharks nei | Squalidae |
|  | Dogfishes and hounds nei | Squalidae, Scyliorhinidae |
|  | Houndsharks,smoothhounds nei | Triakidae |
|  | Nursehound | *Scyliorhinus stellaris* |
|  | Picked dogfish(=spurdog) | *Squalus acanthias* |
|  | Small-spotted catshark | *Scyliorhinus canicula* |
|  | Smooth-hound | *Mustelus mustelus* |
|  | Thornback ray | *Raja clavata* |
|  | Spotted ray | *Raja montagui* |
|  | Small-eyed ray | *Raja microocellata* |
|  | Raja rays nei | Raja spp |
|  | Blonde ray | *Raja brachyura* |
| Miscellaneous aquatic invertebrates | Edible crab | *Cancer pagurus* |
|  | Marine crabs nei | Brachyura |
|  | European lobster | *Homarus gammarus* |
|  | Norway lobster | *Nephrops norvegicus* |
|  | Spinous spider crab | *Maja squinado* |
|  | Velvet swimcrab | *Necora puber* |
|  | Common prawn | *Palaemon serratus* |
|  | Common edible cockle | *Cardium edule* |
|  | Common shrimp | *Crangon crangon* |
|  | Blue mussel | *Mytilus edulis* |
|  | European flat oyster | *Ostrea edulis* |
|  | Great Atlantic scallop | *Pecten maximus* |
|  | Pacific cupped oyster | *Crassostrea gigas* |
|  | Periwinkles nei | Littorina spp. |
|  | Queen scallop | *Aequipecten opercularis* |
|  | Whelk | *Buccinum undatum* |
|  | Variuos shellfish | Crustacea, Mollusca, Echinodermata |
| Squids, cuttlefishes, octopuses | Various squids nei | Loliginidae, Ommastrephidae |
|  | Octopuses, etc. nei | Octopodidae |
|  | Common squids nei | Loligo spp. |
|  | Cuttlefish,bobtail squids nei | Sepiidae, Sepiolidae |
|  | Common cuttlefish | *Sepia officinalis* |
| Miscellaneous demersal fishes | Angler(=Monk) | *Lophius piscatorius* |
|  | Monkfish nei | Lophius spp. |
|  | European conger | *Conger conger* |
|  | Groundfishes nei | Osteichthyes |
|  | Grey gurnard | *Eutrigla gurnardus* |
|  | Gurnards, searobins nei | Triglidae |
|  | Red gurnard | *Chelidonichthys cuculus* |
|  | Tub gurnard | *Chelidonichthys lucerna* |
|  | John dory | *Zeus faber* |
|  | Red mullet | *Mullus barbatus* |
|  | Striped red mullet(=Surmullet) | *Mullus surmuletus* |
|  | Mullets nei | Mugilidae |
