# Supplementary material for: Overfishing and the Replacement of Demersal Finfish by Shellfish: An Example from the English Channel
Source: PLoS One. 2014 Jul 10;9(7):e101506. doi: 10.1371/journal.pone.0101506 (PMC4091961; doi:10.1371/journal.pone.0101506)
Supplement: Supporting Information S1 — Detrended mTL against Detrended FiB with statistical tests. (DOCX) [file pone.0101506.s004.docx]

**Detrended mTL against Detrended FiB**

Further statistical analysis below demonstrate that FiB is strongly related to mTL even after detrending and inter-annual changes in both FiB and MTL are positively related.


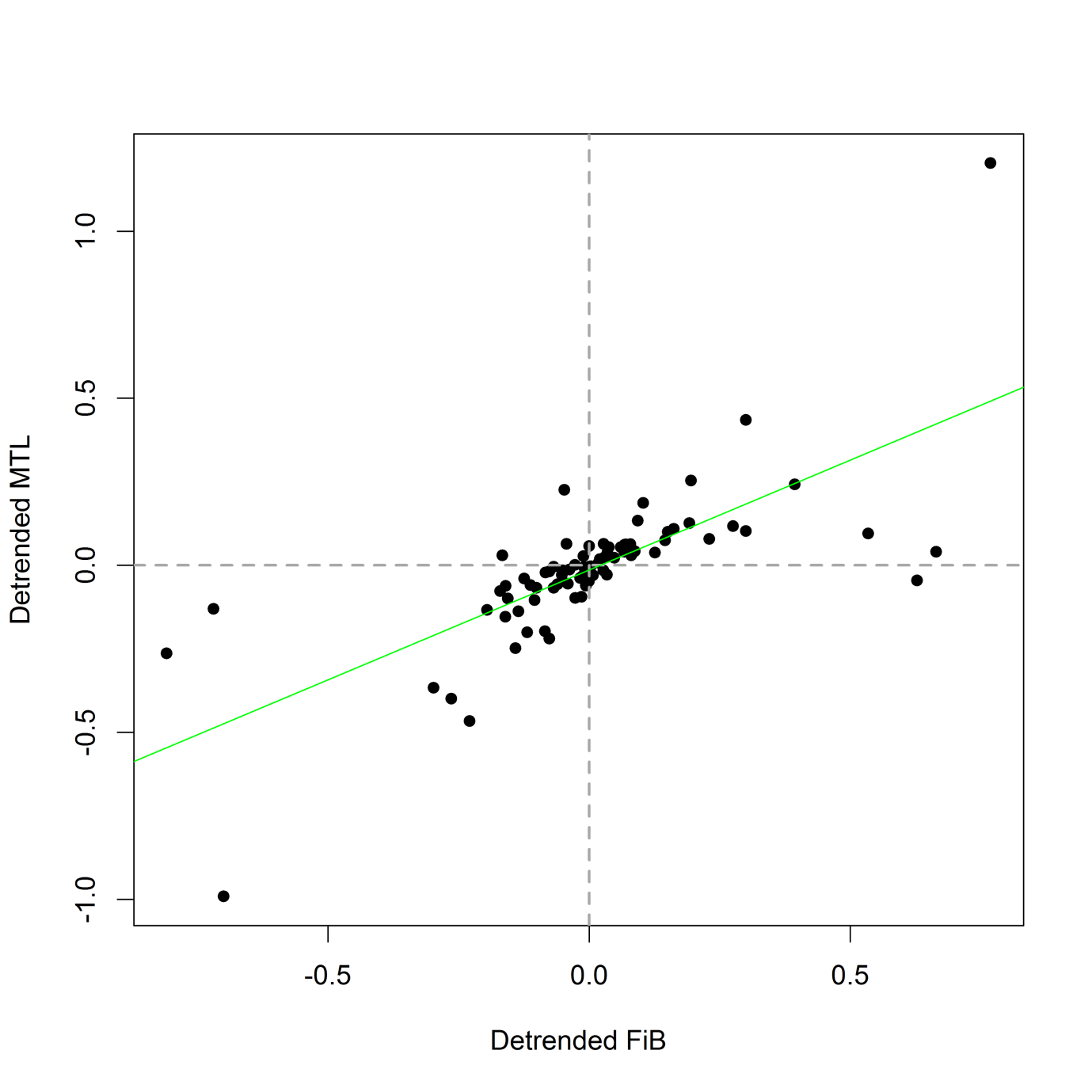


*Analysis of Variance Table Between differenced/detrended MTL and FIB.*
Model 1: dt.mtl ~ 1
Model 2: dt.mtl ~ dt.fib
  Res.Df    RSS Df Sum of Sq      F    Pr(>F)   
1     81 3.9000                                 
2     80 1.9055  1    1.9945 83.732 4.446e-14 ***
---
Signif. codes:  0 ‘***’ 0.001 ‘**’ 0.01 ‘*’ 0.05 ‘.’ 0.1 ‘ ’ 1

*cor.test(dt.mtl,dt.fib) # BEtween detrended/differenced FIB and MTL.*

    Pearson's product-moment correlation

data:  dt.mtl and dt.fib
t = 9.1505, df = 80, p-value = 4.441e-14
alternative hypothesis: true correlation is not equal to 0
95 percent confidence interval:
 0.5896133 0.8069068
sample estimates:
      cor
0.7151205

*Granger Causality Test*

Model 1: mtl ~ Lags(mtl, 1:1) + Lags(fib, 1:1)

Model 2: mtl ~ Lags(mtl, 1:1)

Res.Df Df F Pr(>F)

1. 80
2. 81 -1 3.9339 0.05075 .

---

Signif. Codes: 0 ‘***’ 0.001 ‘**’ 0.01 ‘*’ 0.05 ‘.’ 0.1 ‘’ 1
